# Volumetric analysis of cerebrospinal fluid and brain parenchyma in a patient with hydranencephaly and macrocephaly – case report

**DOI:** 10.3325/cmj.2014.55.388

**Published:** 2014-08

**Authors:** Milan Radoš, Marijan Klarica, Branka Mučić-Pucić, Ines Nikić, Marina Raguž, Valentina Galkowski, Dora Mandić, Darko Orešković

**Affiliations:** 1 Croatian Institute for Brain Research, School of Medicine, University of Zagreb, Zagreb, Croatia; 2 Department of Pharmacology, School of Medicine, University of Zagreb, Zagreb, Croatia; 3 Special Hospital for Chronic Children Diseases, Gornja Bistra, Bistra, Croatia; 4 Department of Molecular Biology, Ruđer Bošković Institute, Zagreb, Croatia; * These authors equally contributed to the study.

## Abstract

The aim of this study was to perform for the first time the intracranial volumetric analysis of cerebrospinal fluid (CSF) and brain parenchyma in the supratentorial and infratentorial space in a 30-year-old female patient with hydranencephaly and macrocephaly. A head scan performed using a 3T magnetic resonance was followed by manual segmentation of the brain parenchyma and CSF on T2 coronal brain sections. The volume of CSF and brain parenchyma was measured separately for the supratentorial and infratentorial space. The total volume of the intracranial space was 3645.5 cm^3^. In the supratentorial space, the volume of CSF was 3375.2 cm^3^ and the volume of brain parenchyma was 80.3 cm^3^. In the infratentorial space, the volume of CSF was 101.3 cm^3^ and the volume of the brain parenchyma was 88.7 cm^3^. In the supratentorial space, there was severe malacia of almost all brain parenchyma with no visible remnants of the choroid plexuses. Infratentorial structures of the brainstem and cerebellum were hypoplastic but completely developed. Since our patient had no choroid plexuses in the supratentorial space and no obstruction between dural sinuses and CSF, development of hydrocephalus and macrocephaly cannot be explained by the classic hypothesis of CSF physiology with secretion, unidirectional circulation, and absorption as its basic postulates. However, the origin and turnover of the enormous amount of intracranial CSF volume, at least 10-fold larger than normal, and the mechanisms of macroencephaly development could be elucidated by the new hypothesis of CSF physiology recently published by our research team.

Hydranencephaly is a difficult and rare developmental disorder that occurs in fewer than 1 of 10 000 newborn children, most likely due to bilateral occlusion of the internal carotid artery between 8 and 12 weeks of pregnancy (1-3). It is often characterized by heavy malacia of the supratentorial structures perfused by the anterior and middle cerebral arteries. Smaller remaining parts of the supratentorial functional parenchyma are located in the area of diencephalic structures and in the areas of the temporal and occipital lobes that are perfused by the posterior cerebral artery (1,3,4). Infratentorial structures are usually morphologically fully developed and functional, and patients are able to perform functions such as suction, swallowing, crying, and moving of the extremities (5-7). This ability and frequently typical neurocranium morphology in newborns hinder the diagnosis of the disease immediately after birth. However, over the next few months an extreme delay in neurological development is observed, often accompanied by hypertensive hydrocephalus and a progressive increase in the head circumference that leads to macrocephaly (head circumference is two standard deviations larger than the average for a certain age). Although most of these patients die within the first few years of life, a small number of them lives for 20 years or longer (6,8-10).

Our patient, aged 30 years, is one of the oldest living patients with hydranencephaly. The clinical picture is dominated by permanent vegetative state with spastic tetraparesis and the morphological picture by extreme macrocephaly. Although in many patients with hydranencephaly detailed qualitative descriptions of intracranial changes have been made, to our knowledge no volumetric analysis of the cerebrospinal fluid and residual parenchyma has been performed separately for the supratentorial and infratentorial spaces. The aim of this article was to quantify the volume reduction of the parenchyma and volume increase of cerebrospinal fluid (CSF) in the supratentorial and infratentorial spaces and to discuss the developmental mechanism of hypertensive hydrocephalus and macrocephaly with respect to dynamic changes of head circumference.

## Case report

We report on the case of a 30-year-old woman with hydranencephaly, in permanent vegetative state, with severe spastic tetraparesis and emphasized macrocephaly. Immediately after birth she had a somewhat larger head circumference but without manifest neurological symptoms. The head circumference was rapidly increasing during the following months, so that at 2.5 months of age it was 45 cm (standard deviation [SD] = 3.43), at 5.5 months 49 cm (SD = 4.52), at 6 months 53 cm (SD = 6.45), at 9 months 57 cm (SD = 8.40), and at the time of the study 75 cm (SD = 14.1).

Signs of spastic tetraparesis started to appear from the 5th month of age and progressed to such a degree that the patient needed continuous medical care. Menstrual cycles are regular but the secondary sex characteristics are incompletely developed. A written informed consent of the legal guardian was obtained before MR exam.

**Figure 1:**
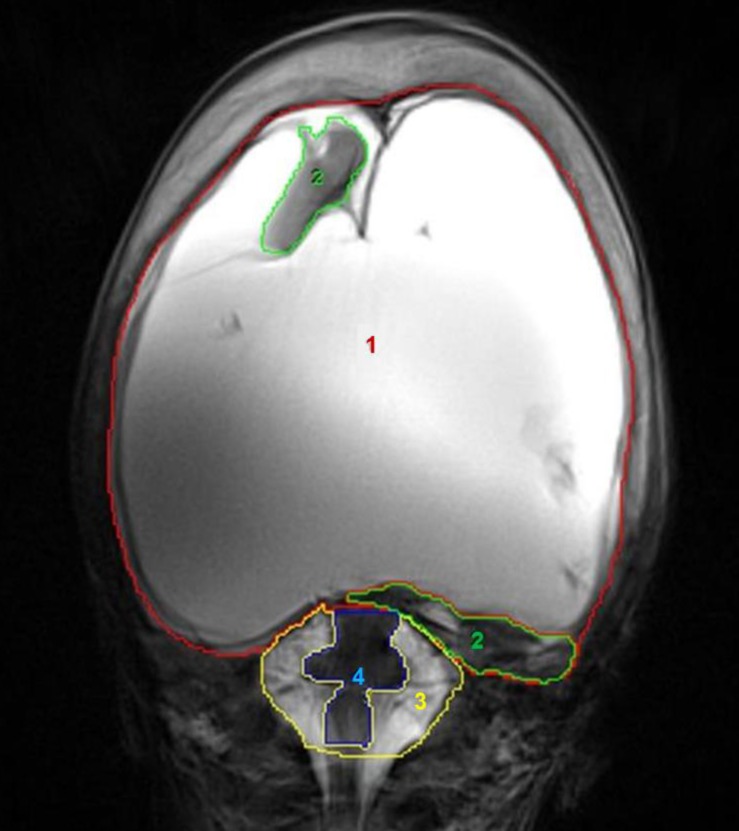
Example of manual segmentation of intracranial parenchyma and cerebrospinal fluid (CSF) in Analyze 8.1 software on T2 coronal slices. 1) Supratentorial CSF space – red line; 2) supratentorial parenchyma – green line; 3) infratentorial CSF space – yellow line; and 4) infratentorial parenchyma – blue line.

Magnetic resonance imaging (MRI) was performed using the 3T MR scanner (Magnetom TrioTim, Siemens, Erlangen, Germany), with a 12-channel Tx/Rc head coil, the only one that has a sufficient diameter to perform head scan in a patient with severe macrocephaly. After the head scan, a coronal T2 sequence (TR = 6000 ms, TE = 106 ms, matrix 320 × 320, FOV = 29.9 × 29.9 cm, voxel size 1 × 1 × 4 mm) was used for a manual segmentation of CSF and brain parenchyma in the supratentorial and infratentorial spaces using the Analyze 8.1 program (Mayo Clinic, Rochester, MN, USA), which measures the volume of the segmented structures in radiological sections (Figure 1).

The analysis of the standard structural MR sequences showed macrocephaly with atypically shaped bones of the neurocranium and a particularly marked anterioposterior diameter. Almost all brain parenchyma of the supratentorial space was affected by malacic changes. A small part of the remaining parenchyma was located in the diencephalon area, the mediobasal part of the left temporal lobe, and the mediobasal part of the occipital lobes, with one small island of parenchyma on the right side, just below the frontal bone. There were no visible remains of the ventricular system or choroid plexuses.

The volumetric analysis showed that the total intracranial volume was greatly increased (3645.5 cm^3^), while the total amount of parenchyma was significantly reduced (169.0 cm^3^), which represents a significant deviation from the average (according to our results in a group of healthy individuals 22.8 ± 0.7 years old, the average intracranial volume was 1512.3 ± 146.5 cm^3^, with a parenchyma volume 1355.7 cm^3^) (11).

The greatest part of the increased intracranial volume was located in the supratentorial space with 3375.2 cm^3^ of CSF, while the brain parenchyma was reduced to only 80.3 cm^3^. In the infratentorial area, the volume of the brainstem and cerebellum parenchyma was 88.7 cm^3^, with the CSF volume being 101.3 cm^3^.

## Discussion

We report on a patient with an extremely large intracranial space, which was primarily the result of an increase in supratentorial space. The supratentorial space was mainly filled with CSF, with only small areas of sustained parenchyma. These results comply with the assumed pathophysiological mechanism of hydranencephaly development due to impaired perfusion in the area supplied by the internal carotid arteries. A small island of sustained brain parenchyma below the frontal bone was primarily perfused by a branch of the right middle meningeal artery and was in no way connected with the rest of the brain parenchyma. Reports of marked macrocephaly are very common in patients with hydranencephaly, but there is no satisfactory pathophysiological explanation for this impressive clinical presentation.

The generally accepted classic hypothesis of CSF pathophisiology has three basic postulates: active secretion of CSF predominantly in the choroid plexuses, unidirectional flow of CSF from the ventricular system to the subarachnoidal area, and resorption of CSF in the dural venous sinuses via arachnoid granulations (11-15). Macrocephaly can only develop if hypertensive hydrocephalus exists before closing of the neurocranial sutures (16). According to this hypothesis, hypertensive hydrocephalus theoretically develops as a consequence of increased secretion of CSF in the choroid plexuses or reduced absorption of CSF in the arachnoid granulations in the dural sinuses, or possibly as a consequence of obstruction of CSF circulation. Since the choroid plexuses are defined as the main place of CSF secretion (17), it seemed logical that their removal (if the CSF pathways are blocked) should prevent hydrocephalus development, and result in the patient’s recovery. For many years, plexectomy was the most popular form of hydrocephalus treatment, but because of universally poor results, it has no place in the current treatment of hydrocephalus (18,19). The introduction of endoscopic method renewed the interest in surgical procedures on the choroid plexuses, especially in the mid 1990s (20-22). Although the results were somewhat better than those obtained using the classic surgical approach, the same problems persisted. The ventricular size was not significantly reduced by choroid plexus coagulation and only 35% of the patients achieved long-term control without cerebrospinal fluid shunts (21). Another study (23) found shunting to be required in 48% of the cases, which was done from one week to thirteen months after the choroid plexus coagulation. All this shows that hydrocephalus can develop even when the plexus has been removed, indicating that its role in the pathophysiology of hydrocephalus is still unclear, but that it clearly does not participate significantly in CSF formation. Despite the resurgence of interest in this approach brought about by each new generation of endoscopes, it has never achieved more than transient acceptance because of its fundamental ineffectiveness.

**Figure 2:**
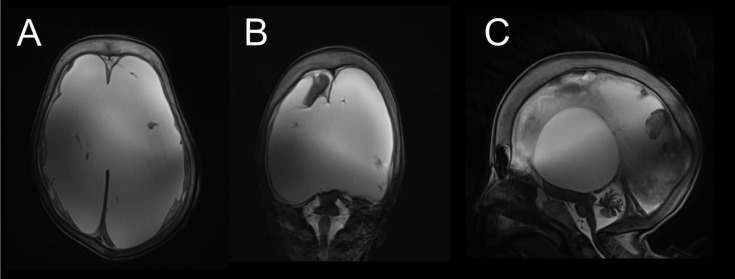
(**A**) Transversal T2 slice across the supratentorial space with almost complete malacia of the cerebral hemispheres and with spared falx and meninges. (**B**) Coronal T2 slice with preserved parts of brain parenchyma in the right frontal lobe, diencephalon, mediobasal left temporal lobe, and brainstem. (**C**) Sagittal T2 slice with visible remnants of the diencephalon, occipital lobe, brainstem, and cerebellum. Marked thickening of the neurocranial bones is visible in all figures.

It is very hard to explain the developmental mechanism of hydrocephalus and macrocephaly in our patient using the basic postulates of the classic hypothesis. Since she had no choroid plexuses in the supratentorial space, the cause of intracranial hypertension and macrocephaly remains unclear. This is especially the case if we take into account that is strongly believed, even taken as a fact (24), that CSF is continuously actively produced by the choroid plexus at a rate of about 500 mL per day. Even more, while in the above mentioned cases it was attempted to stop the hydrocephalus development by removing the choroid plexuses, in our patient the opposite happened: an extensive hydrocephalus, macrocephaly, and severe hydranencephaly (CSF occupies approximately 95% of the cranial cavity) developed without the existence of choroid plexuses in either the lateral or the third ventricle (Figure 1 and 2). Furthermore, it is difficult to explain the constant everyday presence and turnover of such a large amount of CSF, and the maintenance of 30 years of CSF homeostasis.

However, all this can be explained with the new hypothesis of CSF physiology, since it is based primarily on the hydrostatic and osmotic forces at the level of cerebral and spinal capillary network, which has more than 5000 times larger surface than the choroid plexuses (25,26). CNS capillary network is the main area where the volumes of the intracranial fluids and waste products of CNS metabolism are regulated (25,26). This hypothesis has recently been tested by water influx into CSF in aquaporins knockout mice using MRI technique (27), showing that essential for CSF homeostasis was water movement within the pericapillary spaces, rather than choroid plexuses and arachnoid villi.

This new hypothesis (25,26,28) enables us to explain the existence of a permanently large amount of CSF in a case when the brain mass is reduced and supratentorial choroid plexuses do not exist. In this situation, vascularity is still sufficient to permanently maintain the achieved fluid relations inside the cranium. Furthermore, the presented case of hydranencephaly and macrocephaly and other mentioned facts indicate that the key role in the development of hydrocephalus is not played by the choroid plexuses, and that the development of hydrocephalus cannot be explained by a dysfunction in CSF secretion, circulation, and absorption. It seems that the etiology of hydrocephalus, hydranencephaly, and macrocephaly is more complex (28,29) but that it definitely depends on CNS pathophysiological conditions that affect the regulation of fluid volume in the CNS capillary network.

In conclusion, our patient is one of the oldest living patients with hydranencephaly and to our knowledge in such patients no volumetric analysis of the CSF and residual parenchyma has ever been made separately for the supratentorial and infratentorial space. Furthermore, since our patient has no choroid plexuses in the supratentorial space and no obstruction between the dural sinuses and CSF, the development of hydrocephalus and macrocephaly cannot be explained by the classic hypothesis of CSF physiology. However, it can be explained by the new hypothesis of CSF physiology and development of hydrocephalus (25,28-30). This article supports the new hypothesis of CSF physiology (25,26,28) but could also serve as an encouragement to other clinicians to present similar observations to the scientific public.
